# Medium- and Long-Term Effects of COVID-19 in a Population of Patients Admitted to the Intensive Care Unit: Cognitive and Psychological Sequelae and Quality of Life Six Months and One Year after Discharge

**DOI:** 10.3390/healthcare12161624

**Published:** 2024-08-15

**Authors:** Sara Lavolpe, Natascia Beretta, Sofia Bonaldi, Stefano Tronci, Giovanni Albano, Emilio Bombardieri, Paola Merlo

**Affiliations:** 1 Neurology Unit, Humanitas Gavazzeni, 24125 Bergamo, Italy; natascia.beretta@gavazzeni.it (N.B.); sofia.bonaldi@outlook.it (S.B.); stefano.tronci@gavazzeni.it (S.T.);; 2 Intensive Care Unit, Humanitas Gavazzeni, 24125 Bergamo, Italy; giovanni.albano@gavazzeni.it; 3 Scientific Direction, Humanitas Gavazzeni, 24125 Bergamo, Italy

**Keywords:** COVID-19, cognitive sequelae, psychological sequalae, quality of life, cognitive reserve

## Abstract

Most researchers have assessed cognitive functions in post-COVID-19 patients by means of screening tools and found cognitive sequelae in addition to anxiety, stress, depression, and a reduced quality of life (QoL). This study was aimed at investigating cognitive and psychological sequelae in patients admitted to the intensive care unit (ICU) six months (t6) and one year (t12) after discharge from the hospital, the impact of critical illness on well-being and QoL, and the protective role of cognitive reserve (CR). Twenty-three ICU patients underwent an extensive neuropsychological test battery at t6 and t12; a healthy control group underwent the same evaluation. Patient scores were compared with control scores: patients reported significantly lower scores in visual–spatial functions, both at t6 (U = 122; *p* = 0.033) and at t12 (U = 70; *p* = 0.003), and higher levels of anxiety (U = 126; *p* = 0.043) and depression (U = 97; *p* = 0.005) at t6; the levels of anxiety decreased at t12, while only depression symptoms persisted (U = 99.5; *p* = 0.025). Regarding the QoL, patients obtained lower scores in the physical component of QoL, both at t6 (U = 72; *p* = 0.008) and at t12 (U = 56.5; *p* = 0.005). Few and moderate correlations emerged between isolated cognitive functions and CR and the length of hospital stay. The results suggest a prevalent visual–spatial involvement, the medium- and long-term persistence of psychological sequelae, and a reduced QoL in ICU patients.

## 1. Introduction

The severe acute respiratory syndrome coronavirus-2 (SARS-CoV-2), which causes the coronavirus disease 2019 (COVID-19), represented a serious threat for global health over the past few years. Clinical manifestations of COVID-19 encompass a wide range of symptoms, including mild to severe respiratory disorders [1] and extrapulmonary disorders such as gastrointestinal, cardiovascular, psychiatric, and neurological symptoms [2]. Regarding psychiatric symptoms, depression, anxiety, and post-traumatic stress disorder (PTSD) are prevalent among COVID-19 survivors [3], while neurological manifestations range from mild symptoms such as headache, anosmia, and ageusia to more severe complications, including encephalitis, ischemic strokes, delirium, seizures, Guillain–Barrè and Miller Fisher syndrome [4,5]. In Italy, in particular, where the pandemic was most severe, studies highlighted significant cognitive and psychological sequelae in COVID-19 patients, with about 50% of the Italian COVID-19 survivors experiencing cognitive impairment, including memory loss and attention deficits, several months post recovery [6]. Additionally, the prevalence of anxiety and depression in this population was reported to be around 25–40%, with many patients also experiencing symptoms of PTSD [7,8].

Studies reported evidence that SARS-CoV-2 can damage the central nervous system (CNS), leading both to neuroinflammatory responses and neuronal damage and causing hypoxia [9]; thus, neurological sequelae are likely due to brain inflammatory and hypoxic/ischemic damage rather than to the neurotropism of SARS-CoV-2 [10]. To date, four years from the first cases of COVID-19, clinicians are focusing their attention on long-term sequelae, introducing the construct of “Long COVID” or “Post-COVID syndrome”: This refers to symptoms that last longer than three months from the infection and involve fatigue and dyspnea as well as cognitive deficits, “mental fog”, sleep disorders, anxiety, depression, chest and joint pain, palpitations, myalgia, smell and taste dysfunctions, cough, headache, and gastrointestinal and cardiac issues [11,12].

Regarding the cognitive sequelae, a scoping review [13] highlighted that most researchers assessed cognitive functions in post-COVID-19 patients by means of screening tools such as the Mini-Mental State Examination (MMSE) and the Montreal Cognitive Assessment (MoCA) and found lower global scores, with a reduced performance in memory, attention, and executive function sub-tests suggesting a CNS involvement. This finding was confirmed by more thorough studies on cognitive functions carried out by means of an extensive neuropsychological battery [14,15,16,17]. Nevertheless, other studies did not report any cognitive disorders in post-COVID-19 patients [18] and mostly showed persistent emotional disorders such as anxiety, stress, and depression [19]. In this respect, it was suggested that the systemic inflammation resulting from the infection may make patients more prone to persistent depression and then to an associated cognitive impairment [20]. Moreover, there is broad evidence that the infection is also associated with reduced well-being (in both physical and mental areas), post-traumatic stress symptoms, insomnia, and fatigue [15,21]. To our knowledge, few studies have evaluated the cognitive and affective sequelae considering disease severity: In patients with critical illness admitted to the intensive care unit (ICU), delirium, mechanical ventilation, and inflammation were found to be related to cognitive impairment after discharge [22]. A comparison between ICU survivors and general ward survivors highlighted a higher prevalence of microbleeds in the first cohort, but no significant differences in cognitive functioning between the two groups were found [21]. On the other hand, both ICU patients and general ward patients were found to be cognitively more impaired than home-treated patients, especially in the attention, executive function, and memory domains [23,24,25]. The presence of cognitive, affective, and physical sequelae is known as Post-Intensive Care Syndrome (PICS) and has been observed in lots of patients after discharge from the ICU (also not following COVID-19), with symptoms persisting for months or years [26]. Several studies on COVID-19 survivors admitted to the ICU considered a follow-up not more than six months after hospital discharge; fewer studies submitted these patients to more thorough neuropsychological evaluations one year after hospital discharge and highlighted objective cognitive deficits in one-third of such patients, with executive functions, speed of processing, and recognition memory being most impaired [27].

One crucial aspect concerns cognitive reserve (CR), which is a construct intended as the brain’s ability to cope with neurological damage or age-related cognitive decline by using alternative neural networks or cognitive strategies; various factors, such as the level of education, the job type and complexity, and the frequency of leisure activities, contribute to an individual’s CR [28,29]. In this respect, CR can be considered as a protective factor against the cognitive and emotional sequelae derived from COVID-19.

This study was aimed at investigating medium- and long-term cognitive and psychological sequelae, i.e., six months and one year after hospital discharge, in ICU patients without a pre-existing cognitive decline. ICU patients were assumed to exhibit reduced cognitive capabilities, particularly in memory and executive functions, as well as higher levels of anxiety and depression. We were also interested in investigating the medium- and long-term impact of critical illness on well-being and the quality of life (QoL) and the protective role of CR from cognitive and psychological sequelae.

## 2. Materials and Methods

### 2.1. Study Design and Participants

This is an observational, longitudinal case–control study. The medical records of COVID-19 patients admitted to the intensive care unit (ICU) of the Humanitas Gavazzeni Hospital (located in Bergamo, Italy) were examined. Inclusion criteria were as follows: (i) COVID-19 diagnosis by means of polymerase chain reaction (PCR); (ii) presence of acute respiratory distress syndrome (ARDS) requiring intubation and mechanical ventilation in the intensive care unit (ICU); (iii) age between 40 and 75 years; (iv) absence of pre-infection cognitive decline or cognitive disability (defined as Clinical Dementia Rating—CDR [27] score = 0); (v) absence of neurological or psychiatric disorders prior to COVID-19; and (vi) absence of language barriers.

Given the emergency context of the COVID-19 pandemic and the exploratory nature of the study, the sample size was not calculated. Therefore, a total of 85 patients discharged from hospital between March 2020 and March 2021 were contacted by telephone; 23 of them (22 males and 1 female) were enrolled. The other patients were not enrolled because they did not answer on the phone, denied their consent to participate in the study, had cognitive impairment before the infection, or had sequelae requiring long-term hospitalization in specific rehabilitation facilities.

A healthy control group (N = 17; 16 males and 1 female) comprised of individuals who had never before been diagnosed with COVID-19 or with neurological and psychiatric disorders (called “non-COVID-19”) was also selected from the general population.

### 2.2. Procedure

In the enrolment phase, the CDR [30] was administered to a family member of the subjects, with reference to the patient’s pre-infection cognitive status. Patients that obtained a CDR score > 0 were excluded from the final sample.

Cognitive functions, depression, anxiety, and QoL were evaluated six months (±30 days) (t6) and one year (±30 days) (t12) after hospital discharge in ICU patients. CR was only evaluated at t6. Demographic variables, the length of hospital stay, and the length of ICU stay were also recorded. On the other hand, the non-COVID-19 group was only evaluated for cognitive and psychological variables, QoL, and CR.

The t6 assessments started in September 2020 and finished in September 2021; the t12 assessments started in March 2021 and finished in March 2022. The t6 assessments lasted about half an hour and were aimed at collecting clinical, anamnestic, and demographic information before the neuropsychological evaluation. At the end of the t6 evaluations, patients received the date of their follow-up visit; one week before the t12 visit, they were contacted by telephone to remind them of the appointment. The t12 assessments lasted approximately one hour and fifteen minutes. All the assessments were conducted at the outpatient clinics of the Humanitas Gavazzeni Hospital, in calm and interference-free environments. Psychologists with expertise in the neuropsychological area conducted the neuropsychological evaluations. Scores in neuropsychological tests were adjusted for age, education, and gender, according to the Italian published norms, and then converted into equivalent scores (ES), with ES = 0 indicating a “defective” performance, ES = 1 indicating a “borderline” performance, and ES = 2, 3, and 4 indicating a “normal” performance.

### 2.3. Measures

#### 2.3.1. Cognitive Evaluation

The global cognitive status was evaluated by means of the Mini-Mental State Examination (MMSE) [31], a screening tool that assesses various cognitive abilities, including orientation, registration, attention, calculation, recall, and language.

Regarding memory, digit span and Corsi span forward [32] were used to assess short-term verbal and visual–spatial memory. In the digit span task, the examiner presents a sequence of numbers that the participant must repeat in the same order, with the number of digits in each sequence gradually increasing. In the Corsi span task, the participant reproduces a sequence of spatial blocks touched by the examiner in the same order, with the number of blocks also increasing progressively. The Rey’s auditory verbal learning task (RAVLT) [33] was used to assess learning and long-term verbal memory: RAVLT involves presenting a list of 15 words, which the participant must recall immediately after hearing and then again repeatedly over five trials, with a delayed recall after 15 min.

The attention and executive function domains were evaluated as follows: Selective attention was evaluated by means of attentional matrices [34], where participants must cross out target numbers from rows of numbers within three matrices (“5” in matrix I; “2–6” in matrix II; “1,4,9” in matrix III) within a 45 s time limit. The trail-making test (TMT) [35] was used to assess processing speed (part A: the participants connect a series of numbered circles in numerical order—1, 2, 3, etc.—as quickly as possible) and shifting capabilities (part B and B-A: the subjects connect circles alternating between numbers and letters—1, A, 2, B, 3, C, etc.). The Stroop test—short version [36] was administered to assess inhibition capabilities; it consists of three parts: In part one, participants read color names (“red”, “blue”, “green”, etc.) as quickly as possible; in part two, they name the color of a series of colored circles; in part three, participants name the ink color of incongruently printed color names (e.g., the word “red” in blue ink), ignoring the actual word meaning. The verbal working memory was evaluated by means of digit span backward [32], where the examiner presents a sequence of numbers that the subjects must repeat in reverse order. Phonemic and semantic fluencies [37] were used to assess verbal fluency: In phonemic fluency, participants name as many words as possible starting with a specific letter (F-P-L) in 60 s; in semantic fluency, they name as many words as possible from a certain category (car models, animals, and fruits) in 60 s.

Finally, the Rey–Osterrieth complex figure test (ROCF) [38] was administered to investigate visual–spatial constructional functions and planning and monitoring aspects. In this test, participants are asked to copy a bidimensional complex figure.

#### 2.3.2. Depression and Anxiety

Depression symptoms and trait anxiety were evaluated by means of the Beck Depression Inventory-II (BDI-II) [39] and the State-Trait Anxiety Inventory—STAI—Form Y-2 [40], respectively. The BDI-II is a self-report questionnaire used to assess the severity of depression, and it is divided into two scales: Somatic-Affective (including loss of interest, loss of energy, changes in sleep and appetite, agitation, and crying) and Cognitive (including pessimism, guilt, and self-criticism). The STAI Form Y-2 is used to evaluate trait anxiety by asking participants how they generally feel. In both instruments, higher scores suggest higher depression and anxiety symptoms, respectively.

#### 2.3.3. Quality of Life (QoL)

The Sf-12 questionnaire [41], derived from a more extended version, the SF-36 [42], was used to assess the QoL. Two indices can be computed: the physical component summary (PCS), which concerns the physical state, and the mental component summary (MCS), which concerns the mental state. Lower scores in the PCS correspond to a condition of “substantial limitations in self-care and physical, social, and personal activities; significant physical pain; frequent fatigue”; similarly, lower scores in the MCS suggest “frequent psychological distress; social and personal disability due to emotional issues”.

#### 2.3.4. Cognitive Reserve (CR)

CRIq [43] was administered to measure the participants’ CR; in particular, four CRIq scores were computed: CRIq total score, CRIq-education (years of education plus training courses lasting at least six months), CRIq-work (adulthood professions with varying degrees of intellectual involvement and personal responsibility), and CRIq-leisure time (cognitively stimulating tasks carried out during leisure time, distinguished between intellectual activities, social activities, and physical activities).

### 2.4. Statistical Analyses

Data were collected using an electronic data base; statistical analyses were performed using Jamovi 2.3.18. Continuous variables were reported as M—mean (SD—standard deviation). Categorical variables were reported as frequencies (percent frequency). In order to identify any possible outliers, raw scores at cognitive tests were first transformed into Z scores (M = 0; SD = 1): An outlier is defined as a subject whose standardized score falls beyond a threshold of ±3 SD from the mean [44,45].

In case of variables violating the assumption of normal distribution, non-parametric tests were performed. Specifically, comparisons between groups were made by means of the independent samples *t*-test for normally distributed data and of the Mann–Whitney U-test for data violating the normality assumption; as for in-group comparisons, the paired-samples *t*-test or the Wilcoxon-W were performed. The association between variables was assessed by means of Spearman’s correlation coefficient. Statistical significance was set at *p* < 0.05.

### 2.5. Ethical Considerations

Both the patients and the healthy controls expressed their written informed consent before participating in the study. The protocol was approved by the Ethics Committee of our institution and complied with the Helsinki Declaration on human rights.

## 3. Results

### 3.1. Participants

**Figure 1:**
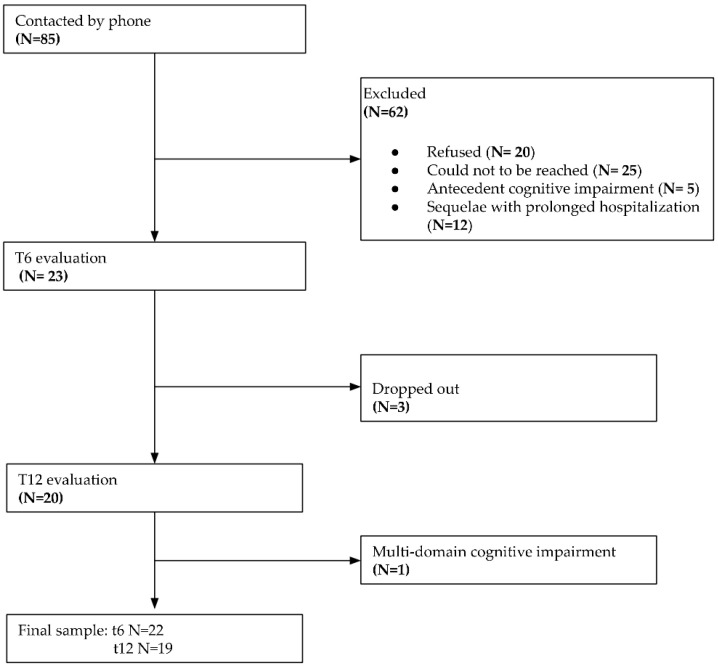
Flowchart of data collection process.

Twenty-three patients were evaluated six months (±30 days) after discharge. Only one patient obtained Z scores lower than 3 SD in the MMSE and in multiple cognitive domains at t6 and t12, indicating a multi-domain cognitive impairment, and was therefore excluded from the final statistical analyses. At the t12 follow-up, three patients dropped out of the study (Figure 1).

Table 1 shows demographic data, the length of hospitalization and ICU stay, CR, and MMSE scores: None of the twenty-two patients received a pathologically correct score in the MMSE at t6 or at t12; as for CR, seven patients (31.8%) showed medium CR, seven (31.8%) showed medium-high CR, and six (27.3%) showed high CR, while only two (9.1%) showed medium-low CR. The difference between the patients and the controls was not significant with respect to the considered variables.

### 3.2. Comparisons between ICU Patients and the Non-COVID Group

The patients received significantly lower scores in the ROCF (U = 122; *p* = 0.033; d = 0.35) six months after discharge when compared with the non-COVID-19 group. A tendency towards significance was observed also in digit span forward (U = 130; *p* = 0.055; d = 0.31) and in the number of errors in the Stroop test (U = 137; *p* = 0.053; d = 0.27), with the non-COVID-19 group performing better than the ICU patients. The difference in the ROCF persisted (U = 70; *p* = 0.003; d = 0.54) at t12; moreover, the patients performed worse than the controls also in Corsi span forward (U = 67.5; *p* = 0.009; d = 0.49), suggesting a prevalent visual–spatial impairment. The tendency towards significance in digit span forward and Stroop test errors, observed at t6, disappeared. No significant differences emerged in the other cognitive domains.

As for the affective state, the patients showed higher levels both of anxiety (U = 126; *p* = 0.043; d = 0.33) and of depression (U = 97; *p* = 0.005; d = 0.48) when compared to the non-COVID-19 group. In particular, 36.3% of the patients showed symptoms of anxiety (22.7% mild, 9.1% moderate, and 4.5% severe), and 18.1% showed symptoms of depression (4.5% mild and 13.6% moderate) six months after discharge. After one year, 21.1% of patients showed persistent anxiety (15.8% mild and 5.3% moderate), and 15.9% showed persistent symptoms of depression (5.3% mild, 5.3% moderate, and 5.3% severe), but only the latter persisted at higher levels in patients when compared to the non-COVID-19 group (U = 99.5; *p* = 0.025; d = 0.38).

As for the QoL, the patients showed lower scores in the physical component of Sf-12 when compared to the non-COVID-group, both at t6 (U = 72; *p* = 0.008; d = 0.50) and at t12 (U = 56.5; *p* = 0.005; d = 0.54).

### 3.3. In-Group Comparisons

Non-parametric paired-samples *t*-tests were performed to compare the patients’ cognitive performance, affective state, and QoL over time, i.e., at t6 and t12: The results summarized in Table 2 show a significant improvement in digit span forward and Stroop time scores and a tendency towards significance in digit backward scores in addition to decreasing levels of anxiety and an improvement in both the physical and mental factor of Sf-12.

### 3.4. Associations between Variables

Correlational analyses showed that the only cognitive variables correlated with the length of hospitalization were phonemic fluency (r = −0.40; *p* = 0.037) and digit span forward (r = −0.60; *p* = 0.003) at t6 and t12, respectively. As for CR, few and moderate positive correlations emerged between semantic fluencies and CRI-education (r = 0.37; *p* = 0.046) and leisure time (r = 0.38; *p* = 0.041) at t6 and between digit span forward and CRI-education at t12 (r = 0.41; *p* = 0.041).

As expected, strong correlations emerged between the patients’ QoL and their affective state at t6, as shown in Table 3. One year (±30 days) after discharge, a significant negative strong correlation persisted but only for mental factor, anxiety, and depression (see Table 4). The length of hospitalization seemed to be negatively correlated with the Sf-12 physical factor at t6 (r = −0.41; *p* = 0.031) but not at t12. CR did not seem to be correlated with the QoL at t6; however, the Sf-12 mental factor at t12 was negatively correlated with CRI-education (r = −0.40; *p* = 0.046) and CRI-work (r = −0.43; *p* = 0.032). Neither the CR indices nor the length of hospitalization and the ICU stay were correlated with anxiety and depression after 6 and 12 months (±30 days).

## 4. Discussion

The main purpose of this study was to investigate the medium- and long-term cognitive and psychological sequelae, i.e., six months and one year after hospital discharge, in COVID-19 patients admitted to the ICU. As for cognitive sequelae, several studies have shown persistent residual deficits in executive functions and in the attention, immediate recall, and working memory domains three, four, or six months after the acute infection, mostly using screening tools [13]. Fewer studies have evaluated long-term cognitive functions [6] more thoroughly and focusing on a specific target population at the same time [21,25]. In this study, ICU patients were thus evaluated by means of an extensive neuropsychological test battery at both six months and one year after hospital discharge: The comparison with healthy subjects, who never suffered from COVID-19 before, only showed persistently reduced visual–spatial capabilities. In particular, the patients received lower visual–spatial constructional scores both six months and one year after discharge and lower short-term visual–spatial memory scores one year after discharge. This evidence is in line with other studies, which reported a prevalent visual–spatial involvement in recovered COVID-19 patients [46,47,48]. At the same time, our patients did not perform worse than the controls in most of the evaluated cognitive domains; similarly, Mattioli and colleagues [19] highlighted the absence of cognitive disorders related to COVID-19 four months after the diagnosis in a population with mild to moderate COVID-19, which points out the sole persistence of severe emotional disorders. On the other hand, our sample only included severely affected patients who received immediate and appropriate treatment in the ICU: This may have prevented the risk of residual neurological and cognitive deficits, as previously noted also in Priftis’ [18] and De Tanti’s [15] works.

The length of hospitalization and the ICU stay as well as certain clinical variables such as disease severity and duration did not seem to affect the patients’ cognitive status [46,49]: The only cognitive functions negatively related to the aforementioned variable in our sample were verbal fluency and short-term verbal memory, even if the patients did not perform worse than the healthy controls in these two cognitive domains. The length of hospitalization was also not related to the presence of anxiety and depression, which, as expected, were more severe in the patients when compared to the healthy subjects six months after discharge. In the long-term, i.e., one year after discharge, the levels of anxiety in our patients decreased, suggesting a possible natural mitigation of the symptoms of anxiety [50]; only the symptoms of depression persisted at higher levels, as confirmed by other studies [9], which found a high prevalence of these symptoms several months after recovery from acute COVID-19. The persistence of symptoms of depression may be attributed to several factors: first, the physiological impacts of COVID-19, including chronic inflammation and neurobiological changes, which may contribute to on-going depression states [51]. Additionally, the socio-economic consequences of the pandemic can have enduring effects on mental health, potentially leading to sustained depression symptoms [52]. This evidence should also be interpreted in consideration of the perceived QoL since the patients showed reduced medium- and long-term physical well-being (evidenced as complaining about “substantial limitations in self-care and physical, social and personal activities; significant physical pain; frequent fatigue”) when compared to the controls (but both physical and mental well-being improved one year after discharge). Moreover, six months after discharge, this variable was related to anxiety, depression, and length of hospitalization. This is in line with the recent literature, which shows persistent mood disorders two to six months after discharge in post-COVID-19 patients in addition to fatigue, sleep disorders, post-traumatic stress, psychological distress, and reduced QoL, regardless of the severity of the acute illness [48,50,53]. All these data highlight the importance of the Long-COVID issue, comprising symptoms lasting longer than 3 months from infection and including autoimmune, hematologic, cardiovascular, endocrine, respiratory, and renal deficits but also neurological (both peripheral and central nervous system) and psychiatric diseases [11]. If, on one hand, there is proven evidence of psychological sequelae of COVID-19, then on the other hand, the cause of these effects is yet not clear: As previously suggested, they could be the result of the action of the virus directly on the CNS, the indirect effects via systemic inflammatory responses to the virus, or the result of psychological stressors such as being infected, stigma, and the experience of being in the ICU [54].

Finally, the other aspect we were interested in was related to CR, defined as “the adaptability (i.e., efficiency, capacity, flexibility) of cognitive processes that helps explain the different susceptibility of cognitive capabilities or day-to-day functions to brain ageing pathology or insult” [28,29]: In particular, we were interested in exploring the protective role of CR from the cognitive and psychological sequelae in ICU survivors. As for cognitive functions, in this study, the level of education and being engaged in stimulating activities were related to improved fluency at t6, while the level of education was related to improved short-term verbal memory capabilities at t12. This is partially in line with De Tanti’s [15] work, where the authors found a significant association of a high CR with the executive functions domain, concluding that CR may positively affect the ability to resume normal social activities. However, our data suggest the existence of occasional relations between CR and few and specific cognitive functions rather than wider cognitive domains. One unexpected result of our study is the absence of a relationship between CR and psychological variables such as anxiety and depression: We expected that patients with higher levels of CR would also show reduced anxiety and depression symptoms. For example, Devita and colleagues [55] found that a higher frequency of cognitively stimulating activities predicts lower levels of depression, anxiety, and obsessive–compulsive symptoms in post-COVID patients. The fact that our sample mostly included patients with medium, medium-high, or high CR, while only two patients had a medium-low CR, may account for the absence of such a predictable result in this study. Thus, CR remains an important protective factor against cognitive and psychological sequelae. An interesting result of this study is the negative correlation of the Sf-12 mental factor with education and occupation as measured by means of CRIq one year after discharge: Higher levels of education and occupation seem to be related to lower perceived mental well-being. While at first this result may appear counterintuitive, it turns out to be interesting in regards to occupation, in particular, if two aspects are considered: First, the higher the complexity and responsibility required by the job, the higher the CR; second, all the patients were still actively employed. The mental engagement and responsibility required by the job and the perceived mental well-being may thus have a mutual influence.

We are aware of some limits of this study. The first concerns the sample size: The analyses were conducted on a small number of participants, with a gender imbalance. However, it is important to consider that these are preliminary data collected during the emergency context of the COVID-19 pandemic, which imposed strict limitations, especially in the initial months of the study. Additionally, the exploratory nature of the study enabled us to gather some interesting preliminary information from a specific group of COVID-19 patients, namely those in the ICU. We intentionally focused on this specific target rather than a broader sample of COVID-19 patients, as seen in other studies with similar sample sizes [15,17]. Additionally, the defined assessment time points, particularly at 6 months, required us to exclude some patients who needed prolonged admission to specific rehabilitation facilities. Other biases that could compromise the generalizability of our data include the three dropouts that further reduced the sample size at t12 and the fact that the variables of interest may have changed over time due to the influence of other uncontrolled variables in the experimental design. Despite these limitations, this longitudinal study adds new knowledge about the cognitive and psychological sequelae of critical illness related to COVID-19, using a comprehensive neuropsychological test battery and incorporating a control group in the same study design. Moreover, although not many significant results emerged regarding CR, this is one of the few studies that have examined this construct in relation to COVID-19.

Finally, this study has several notable implications. It contributes to the ongoing research about the long-term effects of COVID-19, especially among those patients who experienced severe illness and required ICU care. The findings highlight the need for continuous monitoring and support for survivors, with a focus on both cognitive and psychological aspects, while also considering individual differences such as CR in the recovery process. As our understanding of Long-COVID evolves, these results provide a foundation for future research and inform clinical practices aimed at improving long-term outcomes and QoL for affected individuals.

## 5. Conclusions

Currently, one of the most important challenges for specialists is the management of patients with COVID-19 sequelae or symptoms attributable to Long-COVID. However, there are few longitudinal studies on the cognitive and psychological sequelae of COVID-19, particularly concerning patients with critical illness. This longitudinal study focused on the cognitive and affective sequelae in the medium and long term, highlighting a predominant involvement of visual–spatial abilities rather than general cognitive impairment. Finally, the persistent anxiety and depression symptoms and the reduced QoL related to the physical domain are important questions to be considered when treating post-COVID patients.

## Figures and Tables

**Table 1:** Demographic data, length of hospitalization and ICU, CR indexes, and MMSE correct scores.

	Patients (N = 22)	Non-COVID-19 (N = 17)	*p* *
Age	59.3 (7.78) years	60.1 (8.92) years	0.771
Education	14.4 (5.02) years	13.6 (3.73) years	0.576
Hospital stay	47.4 (29.6) days	/	/
ICU stay	21.1 (16.3) days	/	/
CRIq_education	116 (23.00)	110 (12.90)	0.387
CRIq_work	119 (18.40)	118 (20.30)	0.868
CRIq_leisure time	113 (20.20)	116 (21.00)	0.697
CRIq_tot	121 (23.60)	119 (19.60)	0.811
MMSE	28.5 *#* (1.61)	28.7 (1.53)	0.707

* Independent samples *t*-test; *#* MMSE correct score at t6. Note: All the data are shown as mean and standard deviation (SD).

**Table 2:** Non-parametric paired-samples *t*-tests.

	t6 (N = 19)	t12 (N = 19)			
	Mean	Mean	W	*p*	Effect Size
MMSE	28.35 (1.66)	28.34 (1.60)	33.5	0.535	0.015
**Digit span forward**	**5.45 (0.74)**	**5.79 (0.91)**	**21.5**	**0.027**	**−0.59**
Corsi span	5.43 (1.42)	5.09 (1.19)	61.5	0.876	0.352
RAVLT immediate	44.99 (6.77)	48.23 (9.40)	57.5	0.068	−0.395
RAVLT delayed	9.44 (3.07)	10.31 (2.92)	39	0.07	−0.426
Attentional matrices	47.83 (4.24)	47.79 (2.85)	78.5	0.547	0.026
TMT-A	27 (6.74)	24.16 (7.06)	121	0.064	0.415
TMT-B	70.53 (60.78)	58.58 (40.28)	107.5	0.174	0.257
TMT-B-A	41.32 (46.57)	34.58 (36.01)	99	0.286	0.158
Stroop errors	1.45 (3.01)	0.76 (2.39)	35.5	0.222	0.291
**Stroop time**	**20.24 (9.88)**	**17.51 (10.92)**	**138**	**0.012**	**0.614**
Digit span backward	3.98 (1.03)	4.43 (1.12)	31	0.052	−0.483
Phonemic fluencies	33.34 (8.54)	34.47 (7.33)	63	0.168	−0.263
Semantic fluencies	47.37 (6.67)	47.84 (6.48)	65	0.299	−0.15
ROCF	31.89 (2.51)	31.76 (1.48)	77.5	0.528	0.013
**STAI-Y2**	**38.63 (10.96)**	**34.37 (7.79)**	**119.5**	**0.022**	**0.562**
BDI-II	9.32 (8.99)	7.16 (7.99)	93	0.223	0.216
**SF-12 PCS**	**41.2 (10.57)**	**45.5 (7.44)**	**37**	**0.009**	**−0.611**
**SF-12 MCS**	**50 (10.89)**	**54.1 (7.52)**	**50**	**0.036**	**−0.474**

Note: W = Wilcoxon test value; *p* = *p* value. Significant comparisons are highlighted in bold.

**Table 3:** Correlations between Sf-12 factors and anxiety and depression in ICU patients (N = 22) at t6.

		STAI-Y2	BDI-II	SF-12 PCS	SF-12 MCS
**STAI-Y2**	r	—			
	*p*	—			
**BDI-II**	r	**0.825**	—		
	*p*	**<0.001**	—		
**SF-12 PCS**	r	**−0.624**	**−0.628**	—	
	*p*	**0.002**	**0.002**	—	
**SF-12 MCS**	r	**−0.647**	**−0.776**	0.154	—
	*p*	**0.001**	**<0.001**	0.495	—

Note: r = correlation coefficient value; *p* = *p* value. Significant correlations are highlighted in bold.

**Table 4:** Correlations between Sf-12 factors and anxiety and depression in ICU patients (N = 19) at t12.

		STAI-Y2	BDI-II	SF-12 PCS	SF-12 MCS
**STAI-Y2**	r	—			
	*p*	—			
**BDI-II**	r	**0.785**	—		
	*p*	**<0.001**	—		
**SF-12 PCS**	r	−0.329	−0.310	—	
	*p*	0.169	0.197	—	
**SF-12 MCS**	r	**−0.846**	**−0.722**	0.055	—
	*p*	**<0.001**	**<0.001**	0.822	—

Note: r = correlation coefficient value; *p* = *p* value. Significant correlations are highlighted in bold.

## Data Availability

The data presented in this study are available on request from the corresponding author.
